# Supplementary material for: The complete chloroplast genome of Microcycas calocoma (Miq.) A. DC. (Zamiaceae, Cycadales) and evolution in Cycadales
Source: PeerJ. 2020 Jan 13;8:e8305. doi: 10.7717/peerj.8305 (PMC6964695; doi:10.7717/peerj.8305)
Supplement: Supplemental Information 1 — Data set included in phylogenetic and comparative analyses of complete chloroplast DNA sequences. [file peerj-08-8305-s001.docx]

Supplementary Material

# Supplementary Table

**S1.** Data set included in phylogenetic and comparative analyses of complete chloroplast DNA sequences.

|  | Family | Species | Accession No./s |
| --- | --- | --- | --- |
| Ginkgoales | Ginkgoaceae | *Ginkgo biloba* | AB684440 |
| Cycadales | Cycadaceae | *Cycas debaoensis* | KM459003 |
|  |  | *Cycas debaoensis* (Jiang) | KU743927 |
|  |  | *Cycas panzhihuaensis* | NC_031413 |
|  |  | *Cycas revoluta* | NC_020319 |
|  |  | *Cycas taitungensis* | NC_009618 |
|  | Zamiaceae | *Stangeria eriopus* | NC_026041 |
|  |  | *Bowenia serrulata* | NC_026036 |
|  |  | *Zamia fufuracea* | NC_026040 |
|  |  | *Encephalartos lehmannii* | NC_027514 |
|  |  | *Lepidozamia peroffskyana* | NC_027513 |
|  |  | *Macrozamia mountperriensis* | NC_027511 |
|  |  | *Dioon spinolosum* | NC_027512 |
|  |  | *Ceratozamia hildae* | NC_026037 |
|  |  | *Microcycas calocoma* | MN577566 |
